# Porcine brain extract promotes osteogenic differentiation of bone marrow derived mesenchymal stem cells and bone consolidation in a rat distraction osteogenesis model

**DOI:** 10.1371/journal.pone.0187362

**Published:** 2017-11-01

**Authors:** Jia Xu, Yuxin Sun, Tianyi Wu, Bin Wang, Yang Liu, Jinfang Zhang, Wayne Yukwai Lee, Qinglin Kang, Yimin Chai, Gang Li

**Affiliations:** 1 Department of Orthopaedic Surgery, Shanghai Jiao Tong University Affiliated Sixth People’s Hospital, Shanghai, China; 2 Department of Orthopaedics & Traumatology, Stem Cells and Regeneration Laboratory, Li Ka Shing Institute of Health Sciences, Faculty of Medicine, The Chinese University of Hong Kong, Prince of Wales Hospital, Shatin, Hong Kong, China; 3 The CUHK-ACC Space Medicine Centre, The Chinese University of Hong Kong Shenzhen Research Institute, Shenzhen, China; University of Sheffield, UNITED KINGDOM

## Abstract

Distraction osteogenesis (DO) is the gold standard to treat large bone defects, but long consolidation period is a major limitation. Innovative efforts to promote osteogenesis are needed. Porcine brain extract (PBE) was reported to enhance the proliferation and differentiation of multiple primary cells. In this study, we aimed to develop a method for collecting PBE and investigate its effects on osteogenic differentiation of rat bone marrow derived mesenchymal stem cells (rBMSCs) and bone consolidation in a rat DO model. The PBE was collected from neonatal brain tissues of porcine fetus and was used to treat rBMSCs. Following PBE treatment (700 ng/ml), osteogenic differentiation was assessed. Further, we locally injected PBE (7 μg/ml, 100μl) or PBS (100μl) into the gap in a Sprague-Dawley (SD) male rat DO model every three days till termination. X-rays, micro-computed tomography, mechanical testing, histology and immunohischemistry examinations were used to exam the quality of the regenerates. The alkaline phosphatase, calcium deposits, and steogenic markers in the PBE treated rBMSCs were significantly increased. In the rat model, new bone properties of bone volume/total tissue volume and mechanical strength were higher in the PBE treated group. Histological analysis also confirmed more mineralized bone after PBE treatment. The current study reports a standard protocol for PBE collection and demonstrated its positive effects on osteogenic differentiation and bone consolidation in DO. Since the PBE is readily available and very cost effective, PBE may be a potential new bio-source to promote bone formation in patients undergo DO treatment.

## Introduction

Distraction osteogenesis (DO), an effective tool for repairing bone defects and correcting osseous deformities, has been used frequently in clinical practice [1]. However, DO with a bulky external fixator requires a long treatment period and results in a high rate of complications, which hampers its further application in clinic [2, 3]. Thus, novel and effective methods to enhance bone regeneration are needed urgently. Many mechanical and biological attempts have been tried to accelerate bone formation and shorten treatment time, most of which are associated with technical difficulties, high cost, and controversial safety profiles [4, 5].

During the procedure of DO, the proliferation and differentiation of osteoblasts are attributed to mechanical stimuli and resultant factors including the neuromodulatory ones [6, 7]. In the report of Burgess et al [8], some factors from neural tissues were found to be potent mitogens for mesoderm-derived cells, particularly for vascular endothelial cells and some ectoderm-derived cells. Owing to their ability to stimulate fibroblasts proliferation, these growth factors have been named brain fibroblast growth factors (FGFs), which also stimulate bone consolidation [9–11]. The porcine brain, as a source of different growth factors including FGFs, has been traditionally regarded as food supplements to improve brain function by people in some areas of China and Thailand [12]. Moreover, the porcine brain derived peptides could increase the activities of various scavenger enzymes including superoxide dismutase, catalase and glutathione related enzymes for their potential neuroprotective effects [13–16]. Therefore, we introduced a method of collecting porcine brain extract (PBE) in this study, and investigated the effect of PBE on osteogenic differentiation of rat bone marrow derived mesenchymal stem cells (rBMSCs) and bone consolidation in a rat DO model.

## Materials and methods

### Chemicals

All the chemicals used were purchased from Sigma-Aldrich, USA, except where specified.

### Ethics

All of the animals were provided by the Laboratory Animal Research Centre of the Chinese University of Hong Kong. All animal experiments were carried out under the animal license issued by the Hong Kong SAR Government and the approval of the Animal Experimentation Ethics Committee of the Chinese University of Hong Kong (Ref NO. 14-052-MIS).

### Preparation of PBE

Porcine newborn from around 4-month uncomplicated pregnancy was used for PBE preparation. Neonatal brain tissues were collected immediately from the newborn following normal spontaneous vaginal delivery the method of euthanasia for the newborn utilized in our study was fast intraperitoneal injection of dorminal 20% with the dosage of 200 mg/kg body weight. After removing the fat tissues, the remaining tissues were washed in ice-cold 0.9% NaCl to remove all traces of blood. The homogenates were then prepared using phosphate-buffered saline (PBS) with a knife homogenizer and polytron homogenizer. The lipids were then removed by filtering through the 70-μm cell strainer, after which the filtrates were collected and centrifuged at 5000g for 15 min at 4°C, to remove cell debris. Finally, thesupernate fluid were further purified using 0.22-μm filters and termed as original PBE. The protein content in the original PBE was measured using BCSA kit (Thermo Scientific, Rockford, IL, USA) according to the manufacturer’s instruction, and it was70 μg/ml. The original PBE was kept in liquid nitrogen till its further use. For the cell study, original PBE was diluted 100 times before use to a working concentration of 700 ng/ml, while for animal study, a working concentration of 7 μg/ml (100μl) was used.

### Isolation and culture of rBMSCs

Twelve-week-old male Sprague-Dawley (SD) rats were used for rBMSCs isolation. The method of euthanasia for rats was fast intraperitoneal injection of dorminal 20% with the dosage of 50 mg/kg. Bone marrow was flushed out from the bone cavity of the rats and subject to density gradient centrifugation over Lymphoprep^™^ (1.007 g/ml; AXIS-SHIELD, Norway) to obtain the mononuclear cells (MNCs). The MNCs were cultured in Modified Eagle’s Medium of Alpha (α-MEM) (Invitrogen, USA) supplemented with 10% fetal bovine serum (FBS) (Gibco, USA) and 1% penicillin/streptomycin (Gibco, USA) at 37°C with 5% CO2. When colonies were confluent, the cells were trypsinized and re-plated for further expansion and examination. Surface markers including CD31, CD34, CD45, and CD90 (BD Biosciences, USA), were used to determine the purity of MSCs (S1 Fig). The rBMSCs used in this study were between passages 3 and 6 [17].

### Osteogenic differentiation

The rBMSCs were placed in a 12-well plate at a concentration of 5000 cells/cm^2^ and were incubated in the α-MEM at 37°C in a 95% humidified atmosphere of 5% CO2. When over 80% confluence was reached, the medium was replaced with osteogenic induction medium (OIM) containing 1 nM dexamethasone, 50 uM L-ascorbic acid-2-phosphate, and 20 mM *β*-glycerophosphate or PBE in OIM at a working dose of 700 ng/ml. The OIM and α-MEM only were set as positive and negative control, respectively.

### Alkaline phosphatase (ALP) staining

After rBMSCs were treated with α-MEM, OIM, and PBE for 3 days, the cells were equilibrated by ALP buffer (0.1 M NaCl, 0.1 M Tris-HCl, 50 mM MgCl2.6H2O, PH 9.5) for 5 min twice, incubated with ALP substrate solution (5 μl BCIP and 10 μl NBT in l mL ALP buffer) at 37°C in dark for 60 min, after which the reaction was stopped by distilled water and the plate was dried before taking photos.

### Alizarin Red S staining

After 7 days of osteogenic induction, rBMSCs were stained with Alizarin Red S (PH 4.2) for 10 min at room temperature and washed with distilled water. To quantify the mineralization, the monolayer was eluted with 10% cetylpyridinium chloride (CPC), and the absorbance was measured at 570 nm.

### RNA extraction and real-time PCR

At day 3 and day 7 of the osteogenic induction with PBE treatment, the genes associated with osteogenesis were assayed by quantitative real-time PCR. Total cellular RNA was isolated with RNA Mini Kit (Invitrogen) according to the manufacturer’s instructions. The amount of total RNA reverse-transcribed was 500 ng. First-strand cDNA was synthesized with M-MLV reverse transcriptase (Invitrogen). PCR amplification was performed using Step One Plus Real-Time PCR System (Applied Biosystems, USA). Primer sequences of osteogenic markers were listed in Table 1. The relative quantification of gene expression was analyzed with the values of 2^-ΔΔCT^, normalized with GAPDH expression level.

**Table 1:** Primer sequences for quantitative real-time PCR.

Forward primer sequence (5'-3')	Reverse primer sequence (5'-3')	Size (bp)	Gene name
GGACAATGAGATGCGCCC	CACCACCCATGATCACATCG	101	Alkaline phosphatase (ALP)
GCATCGCGCCCCTTATCC	GGCGGTACAGGTCGAGCATA	142	Bone morphogenetic protein 2 (BMP2)
GGAGAGAGCATGACCGATGG	GGGACTTCTTGAGGTTGCCA	184	Collagen type 1α (Col1α)
CGGCAAGTTCAACGGCACAG	GAAGACGCCAGTAGACTCCACGAC	148	Glyceraldehyde-3-phosphate dehydrogenase (GAPDH)
GCATTCTGCCTCTCTGACCT	GGGCTCCAAGTCCATTGTT	133	Osteocalcin (OCN)
AAGGTTGTAGCCCTCGGAGA	TTGAACCTGGCCACTTGGTT	128	Runt-related transcription factor 2 (Runx2)

### Animal surgery and distraction protocol

Twenty 12-week-old SD male rats were used. Before surgery, each animal was placed under general anesthesia with a dosage of 0.2 ml/100g body weight via intraperitoneal injection of a solution of 0.2% (vol/vol) xylazine and 1% (vol/vol) ketamine in PBS. Four rats were housed in each cage. All animals were subjected to a right tibia osteotomy procedure with a closed fracture of fibula. A monolateral external distraction fixator (Xinzhong Company, Tianjin, China) was assembled to fix the osteotomy site. Following surgery, rats were allowed to eat and drink ad libitum. Antibiotic (amoxicillin 1.5 mg/100g weight) was administered intraperitoneally for following 3 days. All rats were randomized equally into two groups: PBS group (n = 10): DO with PBS injection; PBE group (n = 10): DO with PBE injection.

The distraction protocol consisted of three phases: latency phase of 5 days, 10-day active distraction phase (1mm/d, in two equal increments), and a consolidation phase of 6 weeks. During the latency and distraction phase, animals were monitored twice a day, while during the consolidation phase, animals were monitored once a day. From the beginning of consolidation phase, both groups received injection of PBS (100 μl) and PBE (100 μl, 7 μg/ml) into the distraction gap every three days till termination, respectively. All rats received subcutaneous injection of Calcein (10 mg/kg) at the beginning of the consolidation phase, and Xylenol Orange (30 mg/kg) three days before termination. No animal became severely ill or died at any time prior to the experimental endpoint. Four rats in each group were terminated at day 36 after surgery, while the rest were terminated at day 57 after surgery. Bilateral tibias were harvested, strapped free of muscle and processed for further examinations.

### Digital radiographs

Distraction zone was monitored by weekly X-ray from the beginning of consolidation phase using the digital X-Ray machine (MX-20, Faxitron X-Ray Corp., Wheeling, IL, USA) under an exposure time of 6000 ms and a voltage of 32 kv.

### Micro-computed tomography (μCT)

The structural change within the distraction zone at week 3 and 6 after distraction was quantitatively assessed with a high-solution μCT (μCT40, Scanco Medical, Bassersdorf, Switzerland) [18]. Three dimensional (3D) reconstructions of mineralized callus were performed. Histomorphometric analysis was done using sagittal images of the distraction zone. Low- and high- density mineralized callus of distraction zone were reconstructed according to different thresholds (low attenuation = 158, high attenuation = 211) using the established evaluation protocol with small modification [19]. The high-density tissues (211–1000 threshold) represented the newly formed highly mineralized bone, while the low ones (158–211 threshold) represented the newly formed callus. Bone volume/total tissue volume (BV/TV) of each specimen were recorded for analysis.

### Four-point bending mechanical testing

Specimens harvested at week 6 after distraction were subject to mechanical test within 24 hours after termination. The contralateral tibia was tested as an internal control. A four-point bending device (H25KS; Hounsfield Test Equipment Ltd, Salfords, UK) with a 250N load cell was used to test the tibia to failure. The long axis of tibia was placed perpendicular to the blades during the test [20]. The modulus of elasticity (E-modulus), ultimate load, and energy to failure were obtained and analyzed using built-in software (QMAT Professional; Tinius Olsen, Inc., Horsham, PA, USA)[20]. The biomechanical properties of the new bone were expressed as percentages of the contralateral intact bone properties.

### Histology and immunohistochemistry

All specimens were fixed in 10% EDTA formalin for 48 h. Half of them were followed by decalcification in 10% EDTA solution for 3 weeks and embedded into paraffin. 5-μm sections were cut using a rotary microtome (HM 355S, Thermo Fisher Scientific, Inc., Germany) along the long axis in sagittal plane. After deparaffinization, immunohistochemistry staining was done. The other half were managed by gradient alcohol dehydration, xylene defatting, and embedded in methyl methacrylate. Thin (5 μm) and thick (10 μm) sections were cut with the RM2155 hard tissue microtome (Leica, Wetzlar, Germany) along the long axis of distraction zone, respectively. The 5-μm sections were stained with Goldner Trichrome and Von Kossa, while the unstained 10-μm ones were used for dynamic histomorphometric measurements including singled labeled surface (sL.S), double-labeled surface (dL.S), ratio of mineralizing surface to bone surface (MS/BS), mineral apposition rate (MAR), bone formation rate per unit of bone surface (BFR/BS), bone formation rate of bone volume (BFR/BV), and bone formation rate of tissue volume (BFR/TV) with fluorescence microscopy (Leica image analysis system, Q500MC) and OsteoMeasure system (OsteoMetrics Inc., Decatur, GA, USA)[21].

Immunohistochemistry staining was performed using a standard protocol [22]. We incubated paraffin secretions with primary antibodies to rabbit osterix (Osx, Abcam, USA 1:100, ab22552) and osteocalcin (OCN, Santa Cruz, USA 1:100, sc30045) overnight at 4°C. The positive stained cell numbers and area in the whole distraction zone per specimen in three sequential sections (50 μm, 150 μm, and 250 μm) per rat in each group were counted and compared, which were expressed as the percentages of the bone volume.

### Statistical analysis

All quantitative data were analyzed using SPSS 18.0 software for windows (SPSS, Chicago, IL, USA). Mann-Whitney U test with a Bonferroni correction was performed for the comparison of mean values, and P < 0.05 was regarded as statistically significant.

## Results

### PBE promoted osteogenic differentiation of rBMSCs

**Fig 1:**
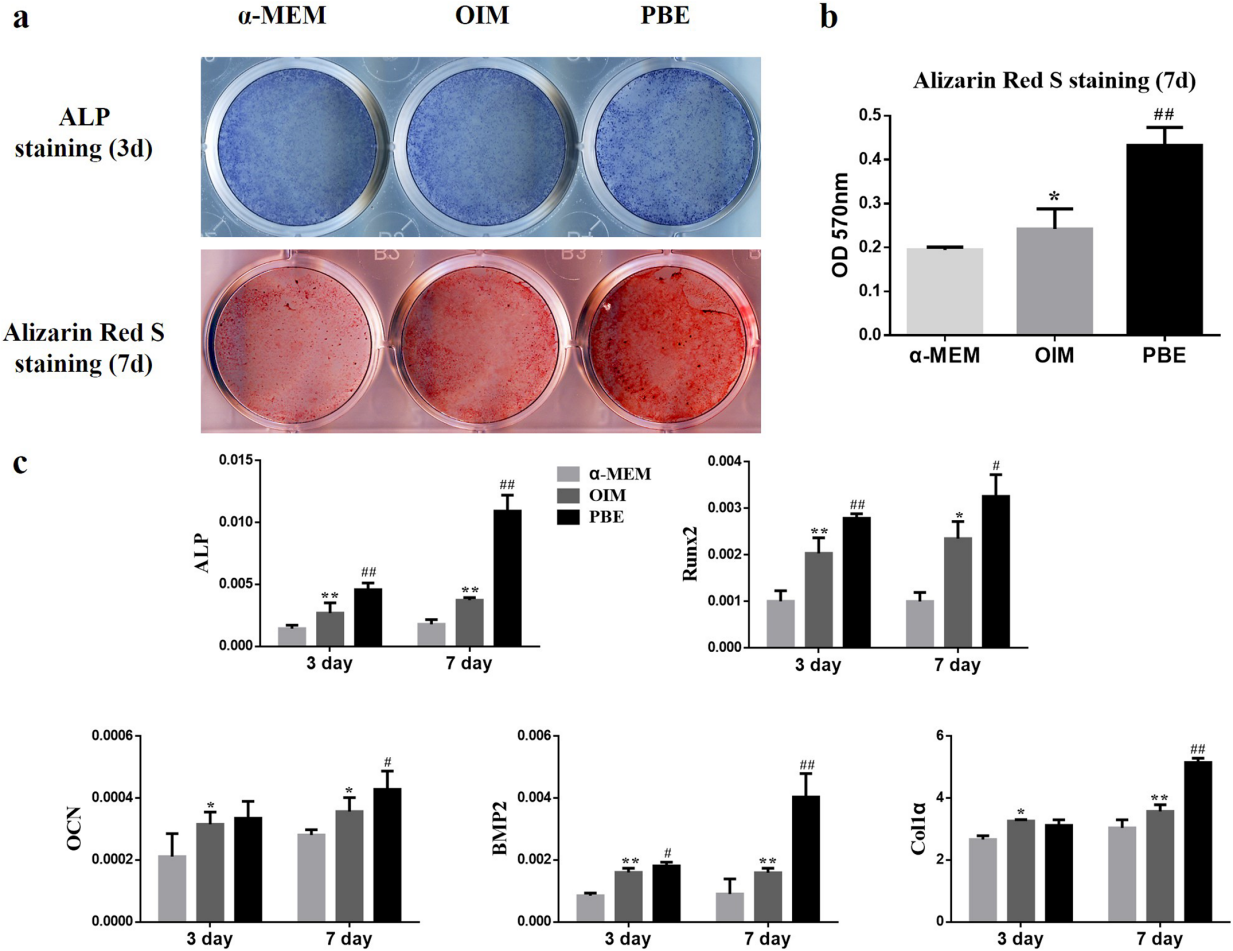
PBE promoted osteogenic differentiation of rBMSCs in vitro. (a) The ALP staining was conducted after 3-day treatment of PBE. The mineralization potential of rBMSCs was tested by Alizarin Red S staining after 7 days of PBE treatment. (b) Alizarin red S concentrations were quantified by absorbance measurement at 570 nM. (c) The genes expression of osteogenesis-related markers was assessed by quantitative real-time PCR after treatment of PBE after osteogenic induction for 3 and 7 days. * p < 0.05, ** p < 0.01, compared to the α-MEM group; # p < 0.05, ## p < 0.01, compared to the OIM group.

Fresh PBE, PBE kept in frozen for 2, 4, and 6 weeks were used for testing the effects on osteogenesis of rBMSCs, and no difference on the effects of rBMSCs osteogenesis was found among the various preparation of PBE. To evaluate the effects of PBE on osteogenesis of rBMSCs, ALP and Alizarin Red S staining were performed at day 3 and day 7, respectively. More mineralized nodule formation could be found in PBE group (Fig 1a). The quantitative results showed PBE significantly increased calcium deposition compared to the α-MEM and OIM group (Fig 1b). Furthermore, significant difference in various osteogenic differentiation-related genes was found in the PBE treatment group after osteogenic induction for 3 days and 7 days. The OCN and Col1α in the PBE treated group were significantly upregulated at day 7, but exhibited no difference compared to the OIM group at day 3 (Fig 1c).

### Radiographic assessment of distraction zone

**Fig 2:**
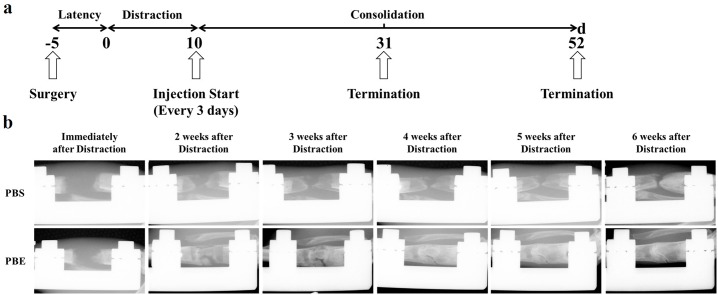
Animal experimental protocol (a) and representative X-rays (b) of distraction regenerate at various time points were present.

**Fig 3:**
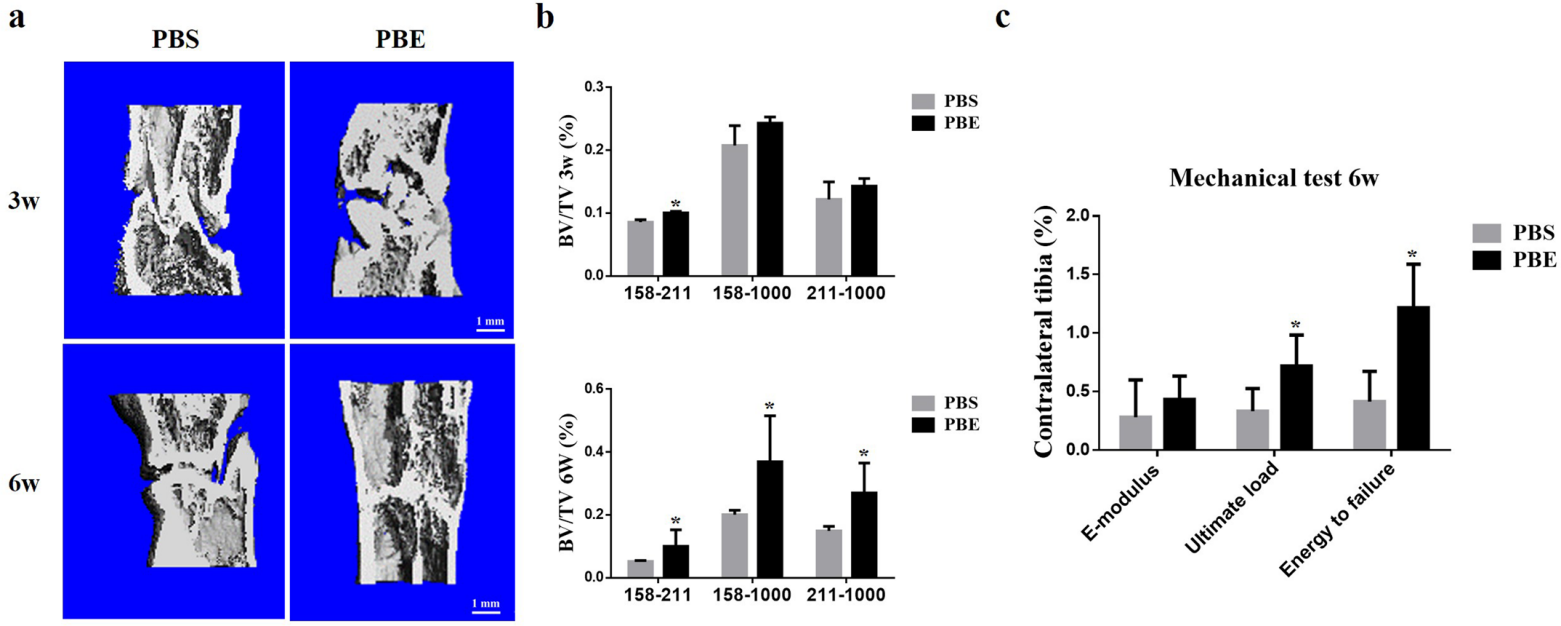
PBE treatment improved the quality of new callus as shown by μCT analysis and mechanical test. (a) 3D μCT images of the tibia distraction zone in the two groups at week 3 and 6. (b) The value of BV/TV at week 3 and 6. (c) Mechanical properties (including E-modulus, ultimate load, and energy to failure) of distraction regenerates. *p < 0.05, compared to the PBS group, n = 4 at week 3; and n = 6 at week 6.

A representative series of X-rays across the time-course of DO show the progression of bone consolidation (Fig 2a and 2b). Little callus was found in the distraction gap immediately after distraction completed in both groups. As went on, significant more callus was observed in the PBE treatment group compared to the PBS group till termination. Similar results were confirmed by μCT examinations at the 3-week and 6-week (Fig 3a). The value of BV/TV at week 3 was significantly increased in 158–211 threshold, while week 6 significant difference was seen in all three thresholds, indicating more new bone consolidation in the PBE treatment group compared to the PBS group (Fig 3b).

### Mechanical testing

The results of four-point bending mechanical testing in the PBE treatment group at week 6 showed a significant improvement in the ultimate load and energy to failure compared to these of the PBS group after normalized with the contralateral intact tibiae. However, there was no significant difference between both groups in E-modulus (Fig 3c).

### Histological analysis

**Fig 4:**
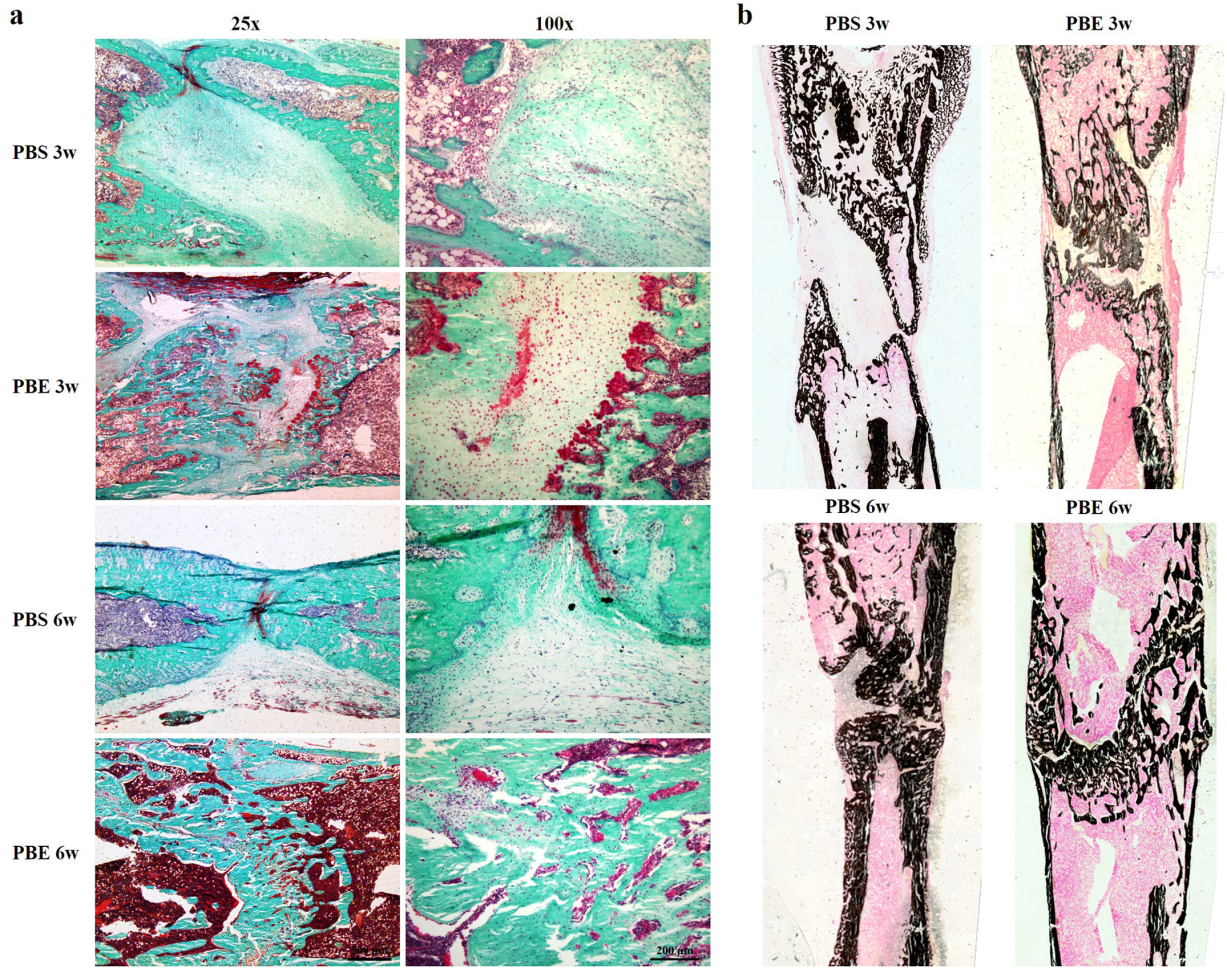
PBE adminstration accelerated new callus consolidation as shown by histological analysis. (a) Representative sections stained with Goldner Trichrome. (b) Von Kossa staining.

**Fig 5:**
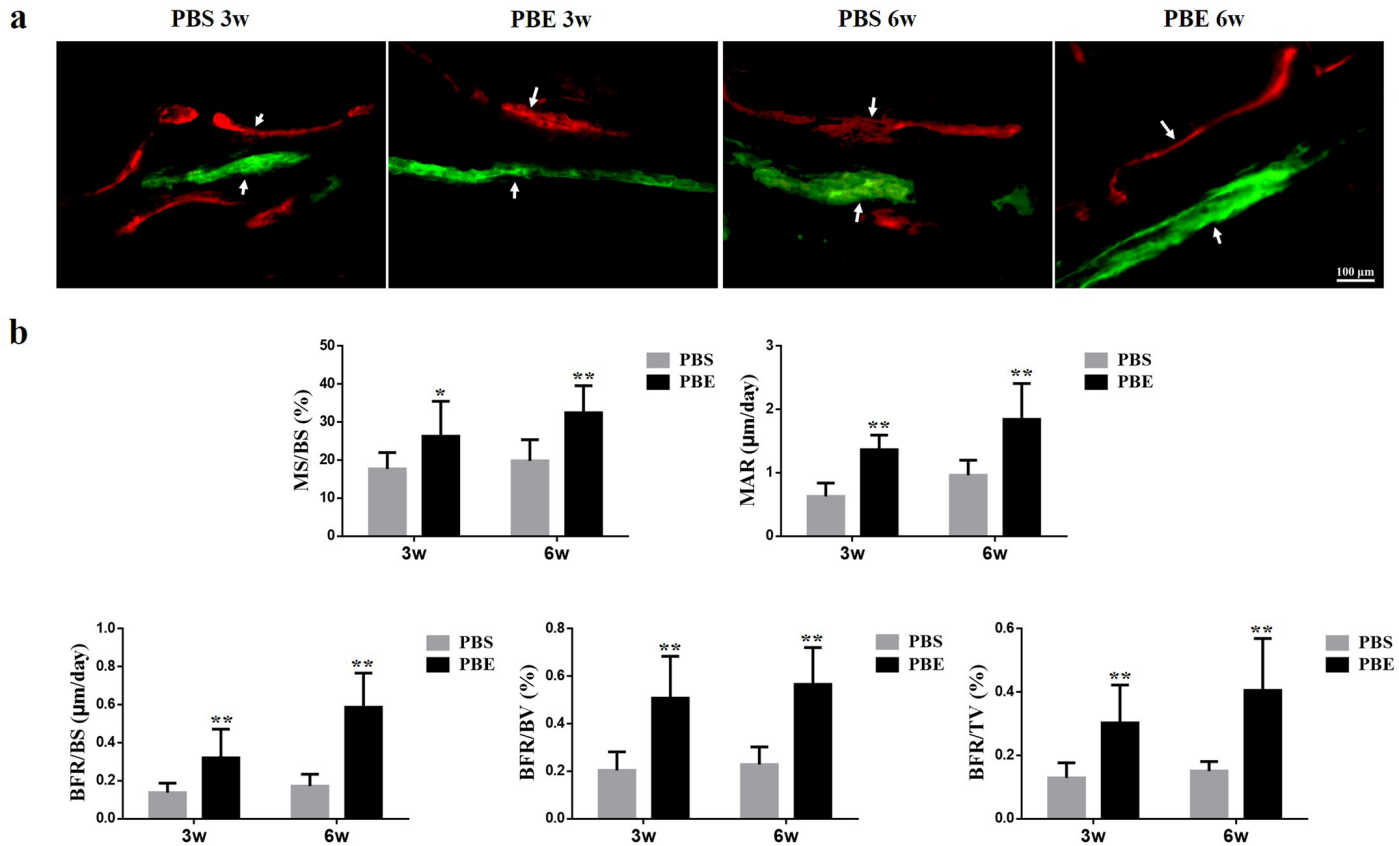
Dynamic histomorphometric measurements showed more quantitative bone formation in the PBE treatment group. (a) Arrows pointed to the Calcein and Xylenol orange labeling in representative images of two groups at week 3 and 6. (b) Quantitative measurements of dynamic histomorphometric parameters of MS/BS, MAR, BFR/BS, BFR/BV, and BFR/TV. *p< 0.05, ** p < 0.01, compared to the PBS group.

**Fig 6:**
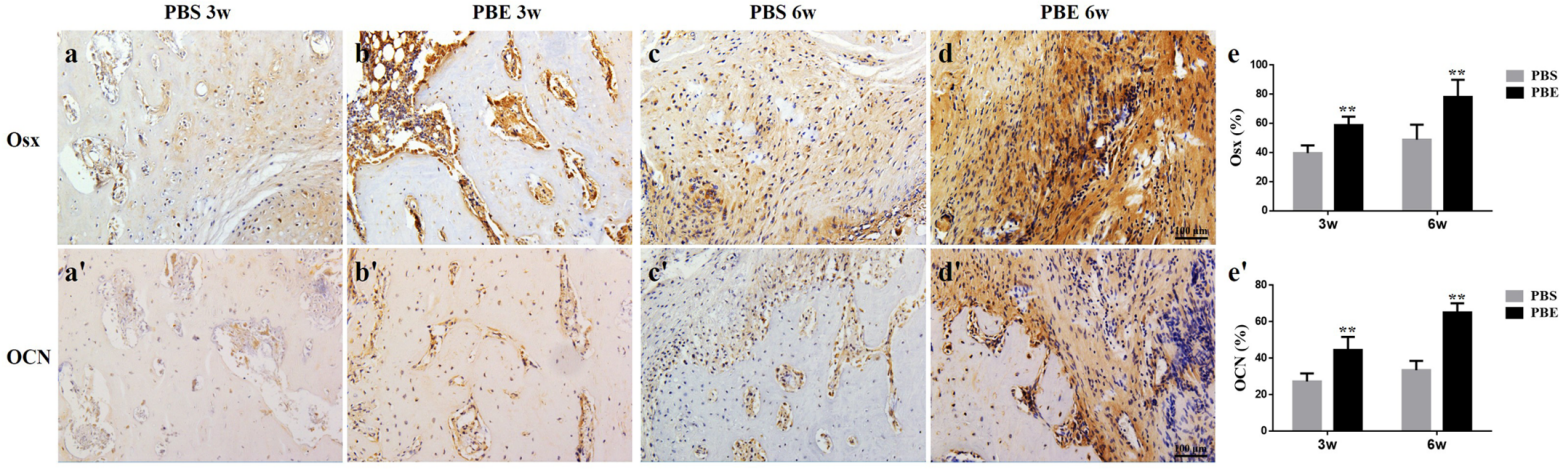
Representative images of immunohistochemical results of Osx (a-e) and OCN (a’-e’) and quantitative analysis of the positive cells in the distraction regenerates. ** p< 0.01, compared to the PBS group.

The representative sections from both groups at week 3 and 6 during consolidation phase stained with Goldner Trichrome and Von Kossa were shown in Fig 4a and 4b. Much more chondrocytes were found in the PBS group than that of the PBE treatment group, especially at week 6, indicating that mineralization of newly formed callus has been accelerated in the PBE treatment group. It was clearly exhibited in the Von Kossa staining that most of new bone had consolidated and the continuity of the cortical bone and bone marrow cavities had almost remodeled in the PBE treatment group at week 6 (Fig 4b). The representative images of dynamic histomorphometric measurements were shown in Fig 5a. The quantitative results demonstrated that the PBE treatment significantly increased MS/BS, MAR, BFR/BS, BFR/BV, and BFR/TV, indicating more mineralized bone formation in the PBE treatment group (Fig 5b). The results of immunohistochemistry staining with Osx and OCN revealed a significant increase in the amounts of positive cells in the distraction gap in the PBE treatment group compared to those in the PBS group at week 3 and 6 (Fig 6). All these results demonstrated that PBE treatment enhanced bone formation during DO.

## Discussion

Among the various methods of stimulating bone formation during DO, biologic stimulations are attractive options. In the present study, we demonstrated that porcine brain extract may have novel potential in augmenting bone formation. This is the first study reporting that PBE has the positive effects on osteogenic differentiation of rBMSCs and bone consolidation in rat DO model.

As a reservoir of a large number of bioactive molecules, the PBE has been shown to have neuroprotective function in the hypoxic-induced pathological animal model and improve the brain functions following their administration [13]. The FGFs derived from the PBE has been well known to regulate proliferation and differentiation of MSCs into mature osteoblasts [10, 23]. Addition of the PBE to culture medium can enhance the proliferation and differentiation of primary cells from rat’s ovary, uterus, and heart [10]. But there was no report on the effect of PBE on differentiation of BMSCs. In present study, we found that PBE treatment promoted osteogenic differentiation of rBMSCs, with increased ALP activity and amounts of calcified nodules formation. The PBE used in the current study was prepared from fresh brain tissues of the newborn pigs, stored in liquid nitrogen till use. Their bioactivities have been confirmed before use in cell culture system.

Other osteogenesis-related genes including ALP, Runx2, OCN, and BMP2, and Col1α, all of which were significantly upregulated following PBE treatment. ALP is required for the proper mineralization of cartilage and bone by hydrolyzing pyrophosphate and generating inorganic phosphate [24]. Runx2 is essential for osteoblast differentiation and plays a key role in chondrocyte maturation. In addition, Runx2 involved in the production of bone matrix proteins [25]. OCN is a marker gene at late stage of osteoblast differentiation, which may explain that there was no difference at day 3 between two groups [26]. BMP2 is produced autogenously when rBMSCs progressed to osteogenic differentiation, and further promotes osteogenic differentiation [27]. As for Col1α, a chondrogenic differentiation marker gene, was significantly upregulated at day 7 by PBE treatment compared to the OIM, while there was no difference at day 3. Since the cartilage-bone transition was a process of endochondral ossification during which bone replaced cartilage gradually under strict regulation of multiple signaling pathways, the late upregulation of Col1α indicated that the chondrogenic differentiation of rBMSCs might also be augmented by PBE [28]. Moreover, the positive numbers of OCN and Osx, which are markers of osteogenesis [22, 29], were remarkably increased in the rat distraction gap after treated with the PBE.

Bone regeneration during DO is regulated by a balance of biochemical and cellular events that stimulate osteoblasts to produce new extracellular collagen matrix then to promote its mineralization [30]. Successful induction of new bone regeneration depends on angiogenesis of the surrounding tissues [31]. The FGFs in PBE are homeostatic factors and mitogens for vascular endothelial cells [8]. Moreover, FGFs play an important role in angiogenesis, vascular permeability, and tissue repair [32]. The basic FGFs are mainly expressed by osteoblasts and MSCs in the newly formed bone during DO [33]. But once distraction phase has ceased, the expression of basic FGFs drops to levels lower than those observed in the distraction phase [34, 35]. Furthermore, in the study of Lee et al. [36], they established a collection of appropriate porcine placental extracts as a food supplement and demonstrated the concentrations of IFN-γ and IL-1 increased in a mice model. In previous studies, systemic administration of IL-1 has been reported to protect rapid bone formation during DO without changing bone mineral density of the intact bone [37]. Also, IFN-γ has been considered as a strong inhibitor of osteoclast differentiation and bone resorption, which in turn promote osteogenesis [38, 39]. Therefore, based on our results, we speculated that PBE contained porcine fetus proteins which might also increase immune activities when applied in vivo.

In addition, according to the study of Eriksson et al [40], the brain derived peptides have neurotrophic effects and promote neurogenesis with unknown mechanism. The PBE used in this study may contain, e.g., neurogenic factors or serotonin, and regulate bone metabolism and remodeling [41, 42]. The mammalian serotonin synthesized in the central nervous system can acts as a neurotransmitter and hormone respectively and stimulates proliferation and differentiation of osteoblasts via the canonical WNT/β-catenin signaling pathway [43, 44]. Whether such porcine brain-derived serotonin may also play an important role in the PBE on bone formation in this study, still need further investigation.

Despite the promising findings in the current study, there are limitations. First, PBE may cause potential immune-responses when applied in vivo. Although we did not see any severe adverse effects in the rats receiving PBE administration, biosafety assessment shall be carried out before PBE clinical application. Also, the future quality of PBE might be controlled using pathogen free pigs. Second, there are multiple factors in PBE, particularly neuronal growth factors, which are important for promoting bone regeneration. But it was unknown which one played the key role during the procedure of bone formation, and since this is a proof-of-concept study, the relationship between neurogenesis and osteogenesis still needs further investigation. Finally, the dose of the PBE (7 μg/ml) may not be the optimal one, and it was injected directly into the distraction regenerate every 3 days till termination, which is not feasible for routine clinical application. The aim of current study was to prove the efficiency, which warrants detailed studies of PBE including the protein concentration, the dose, and other applications in the near future.

In conclusion, we report that PBE promotes osteogenic differentiation of rBMSCs and their local administration enhances bone formation and consolidation in a rat DO model. These findings suggest that porcine brain extract, which is readily available and very cost effective, may be a potential bio-source to be used in augmenting tissue regeneration.

## Supporting information

S1 Fig Cell surface markers of rBMSCs derived from healthy 12-week male rats. Over 97% MSCs expressed the positive markers CD90, and only a few cells (<3%) expressed the negative markers CD45, CD34, and CD31.
(PDF)
