# Supplementary material for: Porcine brain extract promotes osteogenic differentiation of bone marrow derived mesenchymal stem cells and bone consolidation in a rat distraction osteogenesis model
Source: PLoS One. 2017 Nov 1;12(11):e0187362. doi: 10.1371/journal.pone.0187362 (PMC5665543; doi:10.1371/journal.pone.0187362)
Supplement: S1 Fig — Over 97% MSCs expressed the positive markers CD90, and only a few cells (<3%) expressed the negative markers CD45, CD34, and CD31. (PDF) [file pone.0187362.s001.pdf]

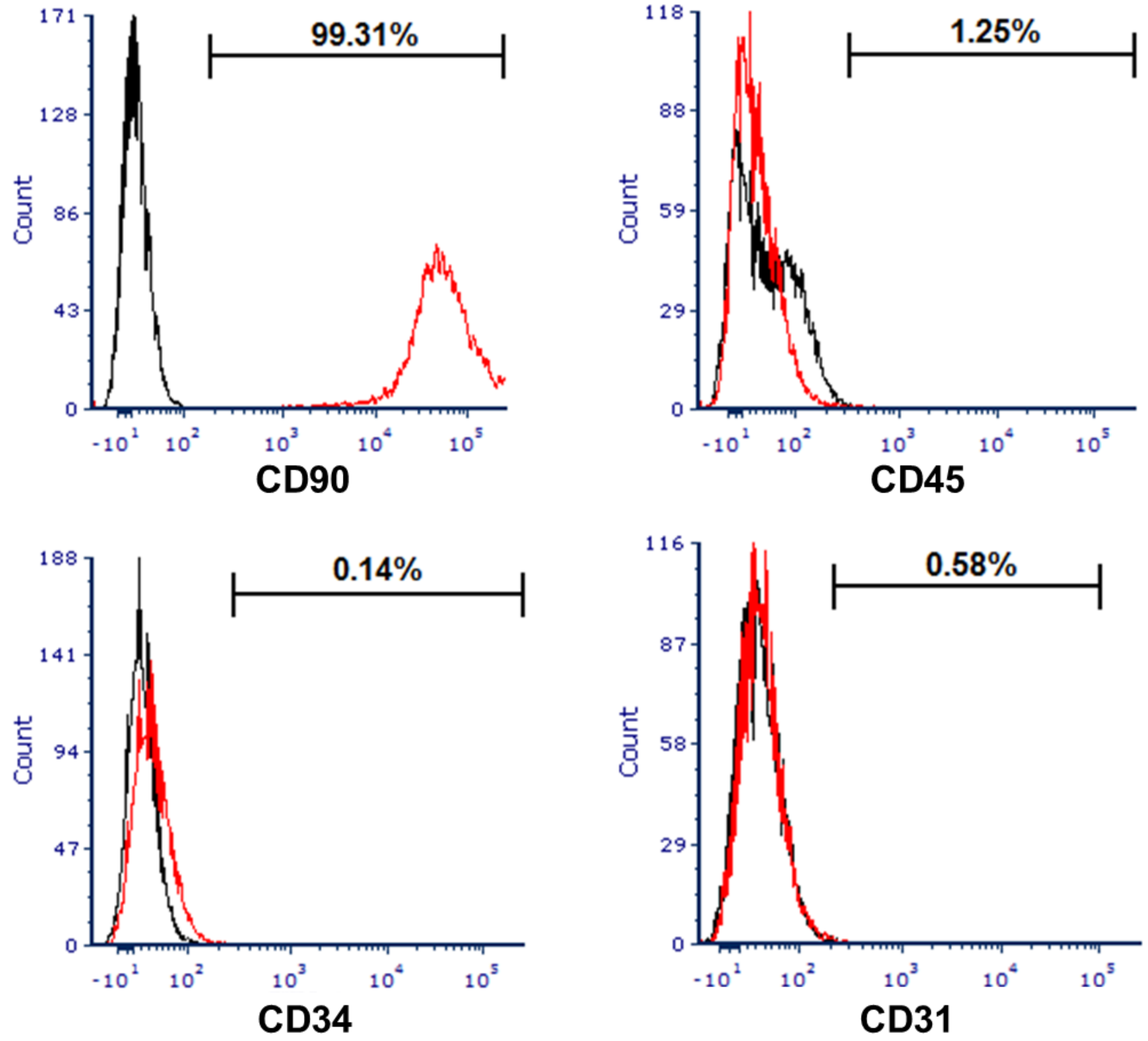

Supplementary Figure 1. Cell surface markers of rBMSCs derived from healthy 12-week male rats. Over 97% MSCs expressed the positive markers CD90, and only a few cells (<3%) expressed the negative markers CD45, CD34, and CD31.
